# Decoding Carbon Dot Purity by Nuclear Magnetic Resonance

**DOI:** 10.1002/anie.6610061

**Published:** 2026-05-01

**Authors:** Yalei Hu, Alberto Bianco

**Affiliations:** ^1^ CNRS, Immunology, Immunopathology and Therapeutic Chemistry, UPR 3572 University of Strasbourg, ISIS France

**Keywords:** carbon materials, dialysis, graphene, NMR

## Abstract

Carbon dots (CDs) have attracted increasing attention in recent years and have been widely explored in many fields. However, several challenges still limit their further development, particularly the unclear atomic structure and fluorescent mechanisms. Addressing these issues requires a thorough purification of crude CD products followed by reliable purity assessment. In this work, we combine dialysis and NMR analysis to evaluate the effectiveness of purification and consequent purity of CDs. Two representative CDs, citric acid‐ethylenediamine‐derived CDs and citric acid‐*p*‐phenylenediamine‐derived CDs were synthesized via hydrothermal treatments. The crude products were first filtered and aliquoted for dialysis. Collected retentates were characterized by microscopy and spectroscopy techniques. With increasing dialysis time, the mass of retentates gradually decreased reaching a steady state. In the NMR spectra, sharps peaks gradually attenuated and eventually disappeared, indicating progressive removal of impurities. Meanwhile, the ratio of integrated broad peaks to sharp peaks increased and reached a plateau with prolonged dialysis. A linear correlation was observed between CD purity and the integrated peak ratios. This work establishes a standardized and (semi‐)quantitative NMR‐based methodology for CD purity assessment, enabling a systematic differentiation and relative quantification of CDs and coexisting small molecular species, thereby addressing a key limitation of previous analyzes.

## Graphical abstract

NMR spectroscopy tracks the removal of molecular and oligomeric impurities in carbon dots, enabling a (semi)quantitative evaluation of their purity through integrated peak analysis.

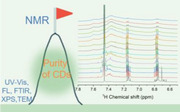

## 1 Introduction

Carbon dots (CDs), a rising class of zero‐dimensional carbon‐based nanomaterials, have been discovered two decades ago [1, 2, 3]. Currently, there are more than 30,000 published papers related to CDs, with an annual increase of around 5000. The publications cover nearly all aspects of CDs, including synthesis, characterization, and applications [4, 5, 6]. CDs were synthesized through various methods such as hydrothermal treatment, microwave method, and pyrolysis of small molecular precursors, biomass, or graphene‐derived materials [7, 8, 9]. The luminescence of CDs extends from violet to near‐infrared, achieving photoluminescence quantum yield of near unity for blue‐, green‐, and red‐emissive CDs [10, 11, 12]. The applications of CDs comprise multiple disciplines, including sensing, bioimaging, cancer therapy, catalysis, energy storage, and anti‐counterfeiting [13, 14, 15].

Although this extensive research on CDs, there are still some gaps. The two most important concerns are the understanding of the fluorescence mechanisms and the precise structure of CDs [16, 17, 18]. About the fluorescence mechanism, several different and even conflicting mechanisms have been proposed, covering quantum confinement effect, surface state effect, cross‐link‐enhanced emission, and molecular fluorescence [19, 20]. Another critical issue is that the atomic structure of CDs is still unclear. Several small‐molecular fluorophores have been found present and clearly identified as structural constituents of the CDs, such as citrazinic acid, 5‐oxo‐1,2,3,5‐tetrahydroimidazo[1,2‐a]pyridine‐7‐carboxylic acid, and 3‐hydroxy‐7,8,13,14‐tetrahydroimidazo[2,3‐k][4,7,11]‐triazaacephenanthrylene‐1,5,10(12H)‐trione [21, 22]. However, the precise structural nature of CDs remains elusive.

To solve the two concerns mentioned above, CDs with extremely high purity are needed [23, 24]. In previous reports, researchers assessed the purity of CDs more by personal skill, and in most cases, it was assumed that the CDs were pure after a certain degree of purification. One study systematically compared the purification of CDs in over 500 publications, finding that over 90% of the isolated CDs were obtained through inadequate purification [25]. Even worse, some authors claimed that as‐prepared CDs did not need any purification, as they were reported to be prepared with high purity. Recently, centrifugation, filtration, dialysis, and chromatography have appeared as commonly used purification methods for CDs, and a combination of two or three of these techniques may work better. These chaotic and complex purification steps largely hinder the elucidation of the structure and fluorescent mechanism of CDs.

After completing the purification, the purity assessment remains also challenging. In the early stage of CD research, the assessment was more empirically determined. In many studies, dialysis was selected for purification, and researchers considered that the dialysis was complete when the dialysate was colorless or non‐fluorescent, as crude products were colorful. However, many small molecules are colorless and non‐fluorescent. In addition, the naked eye is not sensitive enough to determine color and fluorescence. Recently, Prato and colleagues proposed NMR as a powerful method to demonstrate the quality of CDs [26]. They considered that CDs were formed through polymerization and carbonization, which would result in their slower motion in NMR than that of small molecules. Then, NMR spectra of CDs should possess broad peaks rather than sharp peaks indicative of the presence of small molecules. However, this kind of assessment is qualitative, and a semi‐quantitative or quantitative standard is necessary to assess the purity of CDs.

In this work, we propose a combined approach to obtain and assess CDs with high purity, in which filtration and long‐time dialysis were employed for purification, followed by NMR characterization to evaluate CD purity. Two representative CDs were synthesized using aliphatic and aromatic precursors, respectively. During dialysis, the mass of retentates gradually decreased with increasing dialysis time, and eventually reached a minimum, then remained nearly unchanged. At the minimum point, we believe that no more molecular and oligomeric impurities in the sample are left, which could be regarded as “pure” CDs. Based on the ratio of the mass of “pure” CDs to the retentate, the purity of CDs from the different retained fractions could be calculated.

The purity was then quantitatively measured by NMR. In the NMR spectra of retentates at early dialysis periods, both broad and sharp peaks were observed, reflecting the existence of both CDs and molecular/oligomeric impurities. Meanwhile, the ratio of the integrated broad peaks (I_BP_) to the integrated sharp peaks (I_SP_) increased with increasing dialysis time, and eventually reached a maximum, and then remained constant. The calculated purity correlated well with the I_BP_/I_SP_ ratio. For both types of CDs, the NMR‐derived I_BP_/I_SP_‐purity curve was easily plot, thus helping to (semi)quantitative evaluate the purity of CDs and isolate CDs with high purity. This dialysis/NMR combined approach paves the way for CD purification and purity assessment, unlocking their precise structure and fluorescence mechanism, eventually advancing CDs‐related research to the next stage.

## 2 Results and Discussion

**FIGURE 1:**
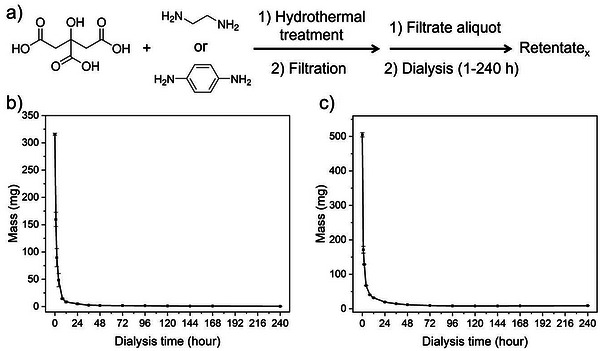
(a) Synthesis and purification steps of CDs; Mass of CE‐CDs (b), and CP‐CDs (c) retentates versus dialysis time. Retentate_x_ means the retained fraction after x hours of dialysis. During dialysis, the external water was replaced seven times per day, maintaining a volume ratio of 1:100 between the solution inside the dialysis membrane and the surrounding medium.

Citric acid‐ethylenediamine‐derived CDs (CE‐CDs) and citric acid‐*p*‐phenylenediamine‐derived CDs (CP‐CDs) were prepared through hydrothermal processes using citric acid (CA)‐ethylenediamine (EDA) and CA‐*p*‐phenylenediamine (PPD) combinations, respectively (Figure 1a). Hydrothermal treatments were carried out at 200 °C for 8 h. As‐prepared crude products were first filtered to remove large‐sized impurities, then dialyzed against water for different durations (1–240 h), and the retentates were collected and lyophilized. For CE‐CDs, the mass of each sample was 315 mg before dialysis (Figure 1b). After 1 h dialysis, the mass of retentate dramatically decreases to around 160 mg. With the dialysis time increasing, the mass of retentate continuously decreases and reaches a minimum after 48 h, remaining almost unchanged up to 240 h. The amounts of purified CE‐CDs after 240 h dialysis are 2 mg, which is a tiny portion (0.63%) of the starting aliquot. This indicates that the CA–EDA system can form CDs, although their proportion may be low under certain conditions, and their yield can be tuned by adjusting reaction parameters. For example, a recent study showed that at elevated temperatures, a larger faction of intermediates undergoes further polymerization and carbonization, resulting in an increased yield of CDs [27]. Photographs of CE‐CD retentates in both solution and solid states under visible light are shown in Figure S1. The color of the solutions gradually changes from brown to nearly colorless.

CP‐CDs showed a similar trend to CE‐CDs (Figure 1c). Increasing the dialysis time, the mass of retentate dramatically decreases again, and reaches a minimum at 120 h. After 120 h dialysis, the recovered mass is 9 mg, corresponding to 1.8% of the starting aliquot (505 mg). The color also gradually changes from dark brown to light brown (Figure S1).

The values of the masses of CE‐CDs and CP‐CDs suggest that at least 48 and 120 h dialysis are required for an adequate purification. The fact that CP‐CDs need longer dialysis time compared to CE‐CDs is likely due to the formation of poorly soluble impurities, which are derived from more hydrophobic PPD than EDA. This also indicates that for a satisfactory purification of CDs derived from different precursors, dialysis time varies. Concerning the long dialysis time required, a previous study demonstrated that using saline solutions can accelerate the dialysis process by reducing the electrostatic interactions between the nanoparticles and the molecular impurities [28]. In addition, the molecular weight cut‐off (MWCO) of the dialysis membrane can also affecting the dialysis performance. Appropriate selection of MWCO can improve the purification efficiency and overall CD purity, with potential implications on final yield depending on the system and conditions.

**FIGURE 2:**
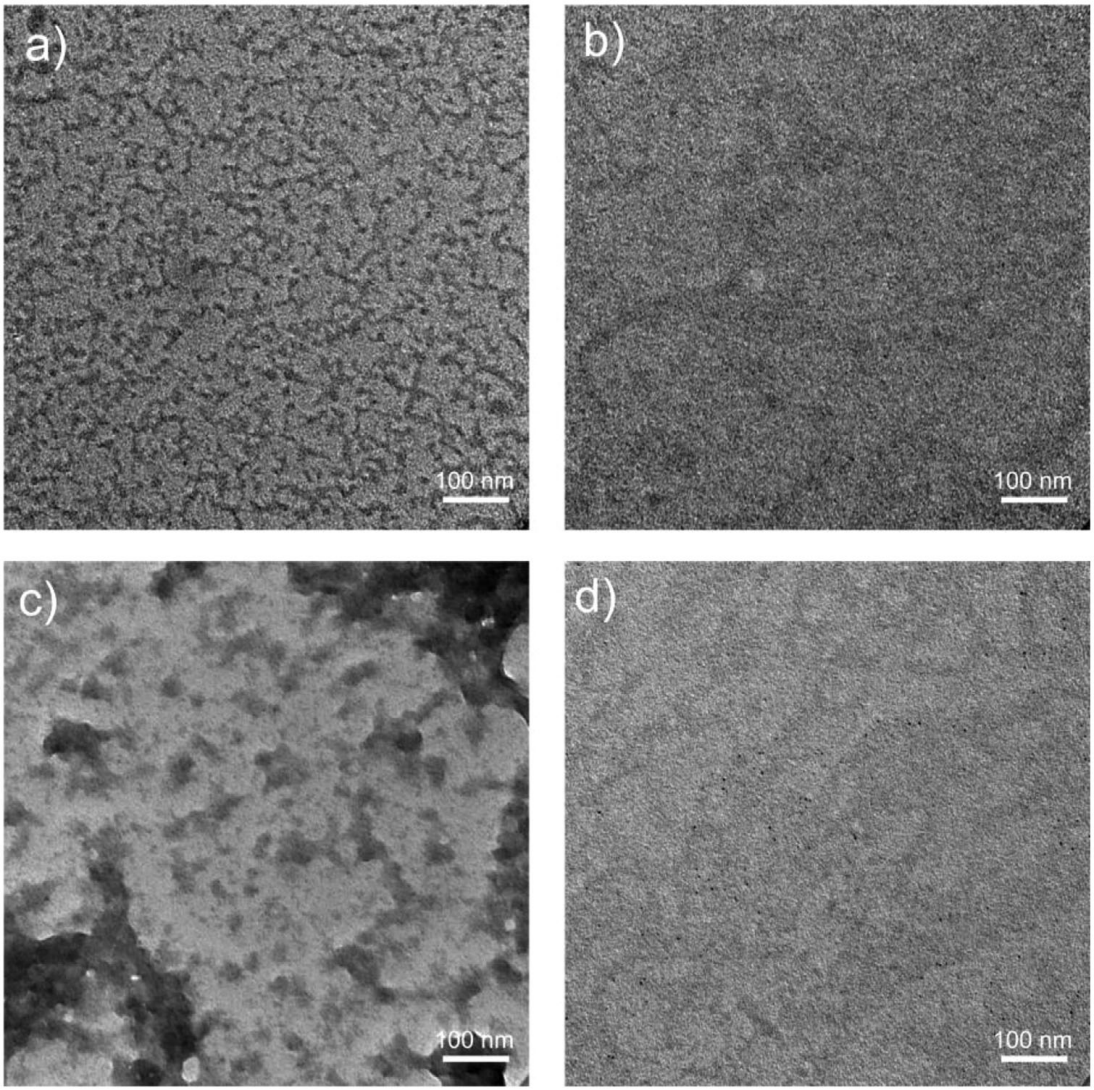
TEM images of CE‐CDs before (a) and after (b) dialysis, and CP‐CDs before (c) and after (d) dialysis.

The morphology of CE‐CDs and CP‐CDs before and after adequate purification was evaluated using transmission electron microscopy (TEM). As shown in Figure 2a, a lot of aggregated amorphous matrix‐like organic residues obscures the observation of CE‐CDs. After 240 h of dialysis, the CDs are clearly observed while no organic impurities are observable (Figure 2b), indicating an adequate purification. The situation is similar for CP‐CDs. Before dialysis, there are abundant amorphous contrasting organic materials (Figure 2c), hampering to see if there are CP‐CDs or not. After 240 h of dialysis, CP‐CDs are very clear, and no impurities are present (Figure 2d). Overall, these TEM images demonstrate that a suitable purification process is essential for the observation of CDs.

**FIGURE 3:**
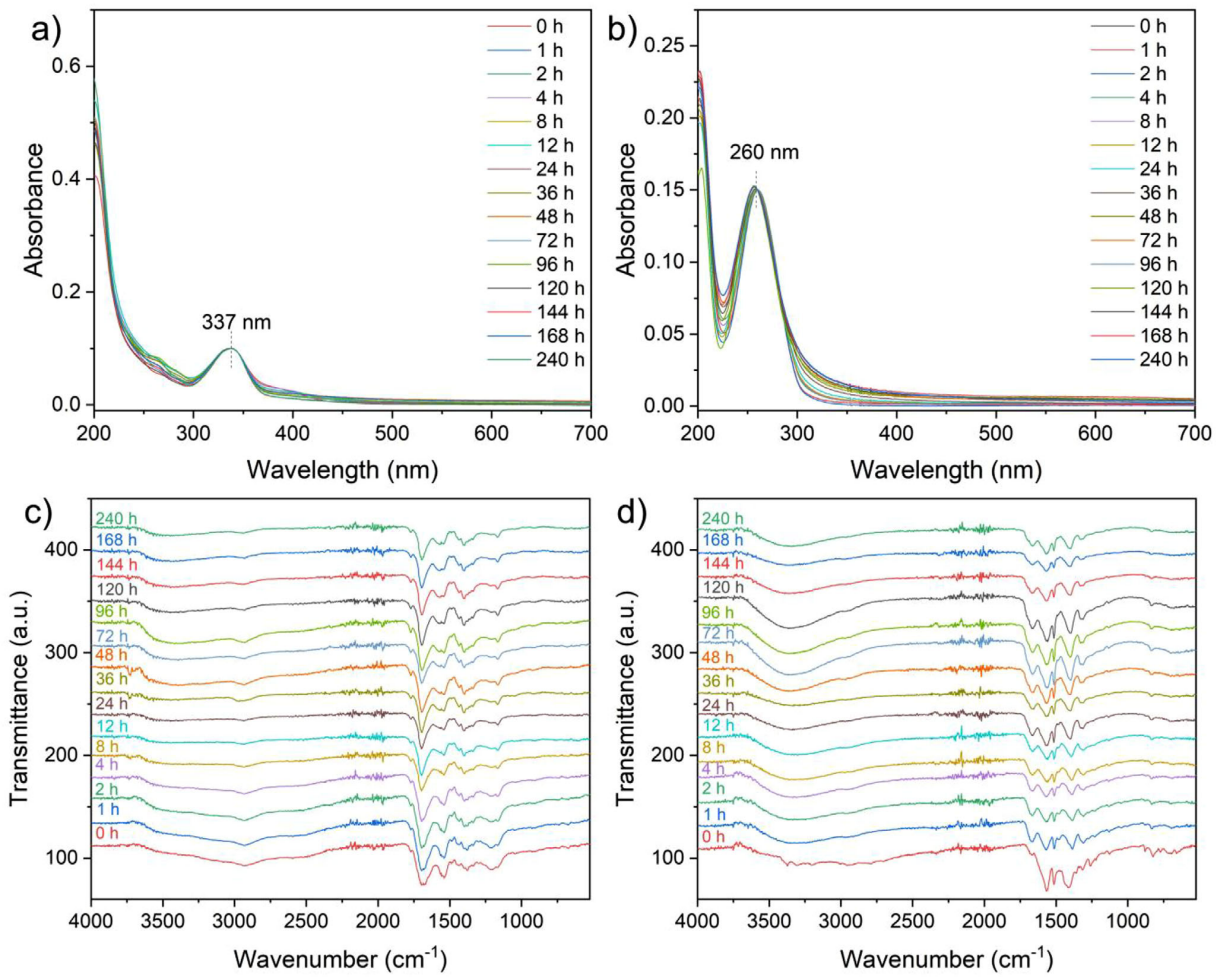
UV–vis absorption spectra of CE‐CDs (a) and CP‐CDs (b) retentates collected at different time points; FTIR spectra of CE‐CDs (c) and CP‐CDs (d) retentates collected at different time points.

UV–vis absorption spectroscopy was then use to analyze the differences among the retentates. All CE‐CD retentates show similar UV–vis absorption spectra, with a main absorption peak at 337 nm and a tail extending to 700 nm (Figure 3a). The absorption band at 337 nm is attributed to the n–π* transitions of C═O and nitrogen‐containing functional groups [29]. These results indicate that the impurities and pure CDs possess very similar structures, which is in agreement with the known bottom‐up growth formation pathway; that is, molecular precursors first undergo dehydration to form oligomers, which then undertake further polymerization and carbonization to CDs [30, 31, 32]. Inside the dialysis membrane, the main impurities correspond to oligomers. Since the oligomers and CDs possess the same moieties, they show nearly identical UV–vis absorption spectra. The retentates of CP‐CDs exhibit a trend similar to that of CE‐CDs, with almost overlapped main absorption peaks at around 260 nm and tails extending to 700 nm (Figure 3b). These results indicate that UV–vis absorption spectroscopy is unable to differentiate CDs from impurities and to prove their purity.

Fluorescence excitation‐emission matrices (EEMs) of CE‐CD and CP‐CD retentates were also recorded. As shown in Figure S2, all CE‐CD retentates exhibit the same excitation‐emission center, with an excitation maximum centered at 340 nm and an emission maximum centered at 430 nm. This observation indicates that CDs and their molecular/oligomeric impurities share again similar emissive centers. The fluorescence EEMs of CP‐CD retentates display the same behavior (Figure S3), which is consistent with the UV–vis results, suggesting that the impurities possess similar structures and thus comparable excitation‐emission characteristics. These results demonstrate that fluorescence analysis is also insufficient to distinguish CDs from the impurities and to evaluate their purity.

Fourier transform infrared (FTIR) spectroscopy was then used to analyze the chemical composition of CE‐CDs and CP‐CDs. Figure 3c displays CE‐CD retentates after different dialysis times exhibiting similar spectra. The broad bands around 3400 cm^−1^ was attributed to the O─H and N─H stretching vibrations [33]. The band at around 2900 cm^−1^ can be assigned to the asymmetric stretching of C─H. In the fingerprint region, there are several peaks at 1750, 1656, 1515, 1350, and 1189 cm^−1^, corresponding to ─COOH, C═C, N─H, C─N, and C─O functions, respectively [34, 35]. It is quite difficult to get any clear insights into the structural differences from FTIR spectra, as all retentates possess nearly identical bands. CP‐CDs retentates exhibit similar infrared characteristics to CE‐CDs (Figure 3d). These results suggest that FTIR is also not powerful enough to identify CDs from impurities.

X‐ray photoelectron spectroscopy (XPS) was subsequently employed to investigate the elemental composition of crude and purified CE‐CDs and CP‐CDs. Both CE‐CDs and CP‐CDs are composed of carbon, nitrogen, and oxygen (Figure S4). Before dialysis, CE‐CDs contain 67.8% carbon, 8.6% nitrogen, and 23.6% oxygen. After 240 h of dialysis, the carbon content increases to 76.1%, whereas the nitrogen content and oxygen content decrease. These changes can be attributed the removal of unreacted precursors and oligomers, which generally contain more heteroatoms than the carbon‐rich CD core formed via dehydration and polymerization [36, 37]. A similar trend was observed for CP‐CDs, where the carbon content also increases after 240 h of dialysis (Figure S4). High‐resolution C 1s spectra of CE‐CDs and CP‐CDs before and after 240 h of dialysis were also collected. As shown in Figure S4c, the relative contribution of C═O/COOH moieties decrease, while the content of C─C increases after dialysis, indicating an increased contribution from the carbon core. A similar trend is visible in C 1s spectra of CP‐CDs (Figure S4d). Although XPS studies confirm the elimination of molecular and oligomeric impurities, it still cannot resolve the purity of CDs.

**FIGURE 4:**
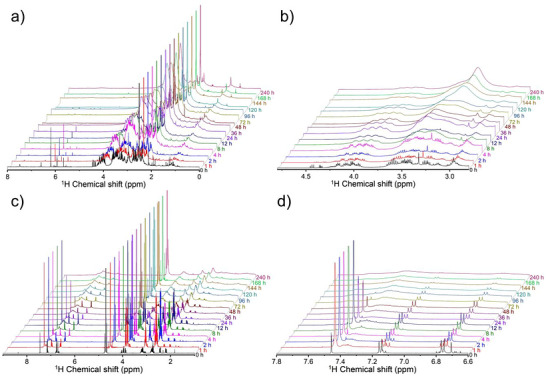
Full and zoom NMR spectra of CE‐CDs in DMSO‐d6 (a and b) and CP‐CDs in D_2_O (c and d) retentates collected at different time points. The individual subfigures are present into Supporting Information for higher readability (Figures S6–S9).

NMR is the key technique for analyzing the structures of small molecules and polymers, and it has been used few times to verify the chemical structure of CDs [38, 39]. Here, it was employed to investigate the difference among the CD retentates collected during the dialysis process and to evaluate their purity. As shown in Figure 4a, b, both sharp and broad resonance peaks are observed for the different CE‐CDs retentates. In the aromatic region (δ 5–8 ppm), sharp peaks gradually decreased in intensity with the increasing dialysis time and eventually disappear after 48 h dialysis, indicating the removal of all molecular and oligomeric impurities. In the aliphatic region (δ 2.8–4.5 ppm), broad peaks with multiple superimposed sharp underneath‐peaks are detectable, originating from contributions of both CD cores and the impurities. Like parasite aromatic sharp resonances, these fine sub‐peaks gradually attenuate and completely vanish after 48 h dialysis. The disappearance of these peaks suggests that CE‐CDs are adequately purified, leaving only the intrinsic broad peaks of CDs. The lack of detectable broad signals in the aromatic region is likely due to the limited carbonization degree and the condensed nature of the aromatic domains, in agreement with a previous literature report [28]. Concerning impurities, Otyepka et al. reported that 5‐oxo‐1,2,3,5‐tetrahydroimidazo[1,2‐a]pyridine‐7‐carboxylic acid was the thermodynamically most stable by‐product in CA‐EDA system [40]. They also identified several new reaction intermediates and pathways through a combination of theoretical calculations, NMR, and matrix‐assisted laser desorption ionization time‐of‐flight mass spectrometry, which provided valuable insights into impurity identification in CD synthesis.

For the CP‐CD retentates (Figure 4c, d), similar evolution can be observed in both the aromatic and aliphatic regions. However, the sharp peaks (δ 7.43–7.50 ppm) persist longer and completely disappear only after 120 h of dialysis, demonstrating that a longer purification time is necessary to fully remove the impurities in this type of dots. This difference likely arises again from the low solubility of PPD precursors, and a poor solubility inherited by PPD‐derived oligomers, thereby prolonging their removal time during dialysis in water. These findings imply that the purification conditions, such as dialysis time or method, should be optimized based on the precursor‐dependent properties. In addition, the purity of CP‐CDs was further confirmed by the diffusion ordered spectroscopy (DOSY). By comparing the DOSY spectra of CP‐CDs retentates at 0 h (Figure S5a) and 240 h (Figure S5b), it is clear that CP‐CDs at 240 h are not contaminated by molecular impurities. Their diffusion coefficients are comparable to previous reported CDs [28].

**FIGURE 5:**
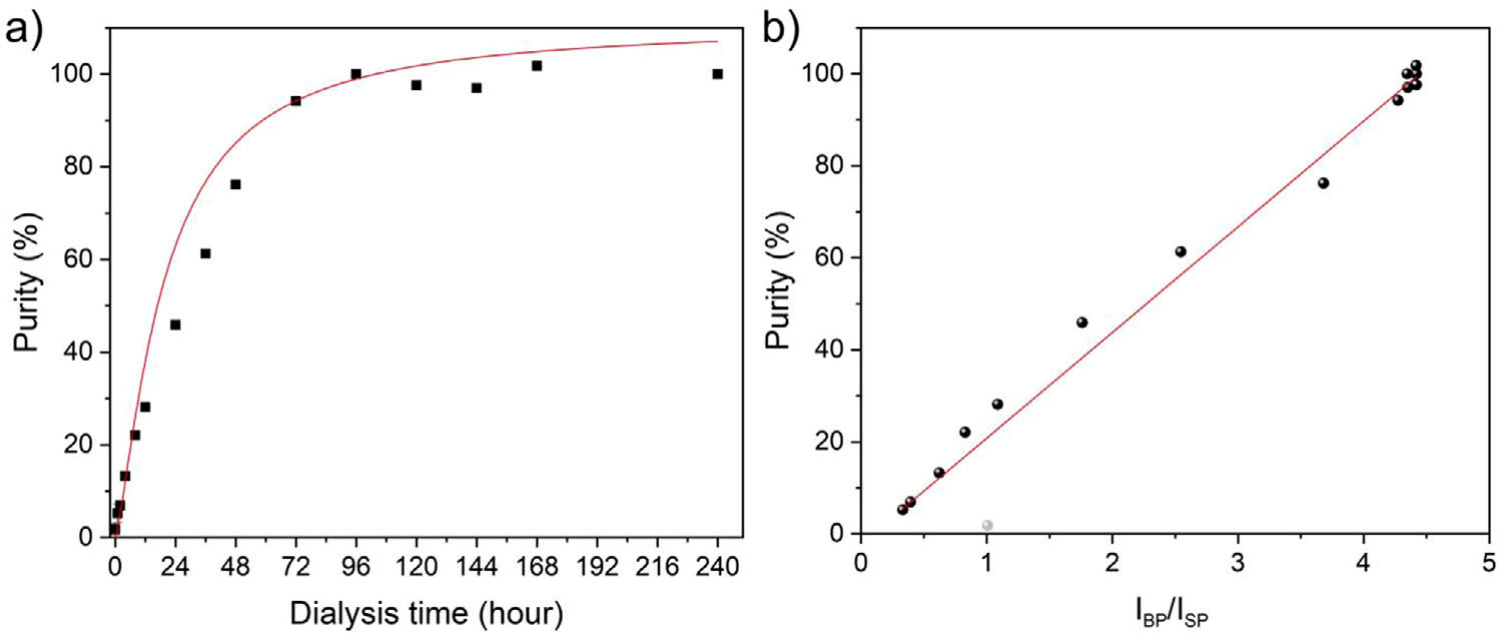
(a) Purity of CP‐CDs retentates versus dialysis time; (b) Purity of CP‐CDs retentates versus I_BP_/I_SP_. An out‐of‐range data point (grey dot) is observed, that we attributed to signal partial saturation caused by high concentration.

From the NMR spectra, the point at which CDs are adequately purified can be visually identified. However, a concern remains regarding how to quantitatively assess CD purity at the different purification phases. From Figure 4c, d, we can see that the sharp impurity signal at δ 7.43–7.50 ppm gradually decreases in intensity, whereas the broad the resonance peaks attributed to the carbon core at δ 7.25–7.43 ppm remains essentially unchanged with increasing dialysis time. Based on this ascertainment, the ratio between the integrated broad peak (I_BP_, δ 7.25–7.43 ppm) and the integrated most intense sharp peak (I_SP_, δ 7.43–7.50 ppm) can be treated as a relative purity indicator. The purity of the retentate at each dialysis stage can be estimated as the ratio of the final purified product mass to the mass of the retentate at each dialysis time point (Figure 5a). By combining these measured I_BP_/I_SP_ values with the purity data, a scatter plot of purity versus I_BP_/I_SP_ can be created (Figure 5b). A strong linear correlation is observed, suggesting that NMR can be used to quantify the purity of CDs. An additional signal in the aliphatic region (δ 3.80–4.20 ppm) was also integrated, and a scatter plot of purity versus I_7.25–7.43_/I_3.80–4.20_ is shown in Figure S10. A similar trend was observed, further supporting the correlation between signal intensities. Such purity‐assessment curves can be calculated for other types of CDs to evaluate their purity.

## 3 Conclusion

In summary, we investigated the purification process of CDs derived from aliphatic (CA‐EDA) and aromatic (CA‐PPD) precursors and evaluated their purity. Both CDs were synthesized via a hydrothermal treatment and subsequently purified through filtration and dialysis. During the dialysis, the mass of the retentate progressively decreased and eventually reached a constant value. The structural and photophysical properties of the retentates were studied by combining microscopy and spectroscopy techniques (e.g., TEM, UV–vis, fluorescence spectroscopy, FTIR, XPS, and NMR). The obtained results show that UV–vis, fluorescence spectroscopy, FTIR, and XPS are insufficient to prove the purity of CDs, whereas NMR enables the direct assessment of the residual molecular and oligomeric impurities. In contrast to prior studies where NMR was primarily used qualitatively, we demonstrate that NMR can be developed into a (semi‐)quantitative analytical platform for resolving the compositional complexity of CDs by correlating the relative purity of the samples with the integrated intensity ratio of broad CD‐related signals to sharp impurity‐related signals in the NMR spectra. The proposed NMR‐based purity validation strategy offers a practical approach to guide CD purification, advance their structural elucidation, and potentially facilitate CD commercialization. Although NMR is a powerful tool for the purity assessment of CDs, its detection limit is generally in the micromolar to millimolar range. Therefore, trace amounts of molecular species present at lower concentrations may not be detectable. In future studies, techniques such as high‐performance liquid chromatography and mass spectrometry could be introduced to provide further insight into the molecular weight distribution and the presence of low‐molecular‐weight species.

## Conflicts of Interest

The authors declare no conflicts of interest.

## Supporting information




**Supporting File**: anie72482‐sup‐0001‐SuppMat.docx.

## Data Availability

The data supporting the findings are available from the corresponding authors upon reasonable request.
