# Supplementary material for: Decoding Carbon Dot Purity by Nuclear Magnetic Resonance
Source: Angew Chem Int Ed Engl. 2026 May 1;65(25):e6610061. doi: 10.1002/anie.6610061 (PMC13266933; doi:10.1002/anie.6610061)
Supplement: Supplementary file 1 — Supporting File: anie72482‐sup‐0001‐SuppMat.docx. [file ANIE-65-e6610061-s001.docx]

Decoding Carbon Dot Purity by Nuclear Magnetic Resonance

Yalei Hu,* Alberto Bianco*

CNRS, Immunology, Immunopathology and Therapeutic Chemistry, UPR 3572, University of Strasbourg, ISIS, 67000 Strasbourg, France

Supporting Information

**Experimental**

*Materials*

Citric acid (CA) was purchased from VWR. Ethylenediamine (EDA) and *p*-phenylenediamine (PPD) were acquired from Thermos Scientific and Sigma, respectively. Dimethyl sulfoxide-d6 and deuterium oxide were obtained from Eurisotop. Dialysis membrane (MWCO: 1 kD) was purchased from Repligen Corporation. All chemicals were used as obtained. All aqueous solutions were prepared by using deionized water obtained from a Milli-Q water purification system.

*Synthesis and purification of CDs*

For CA and EDA derived CDs, 5 g CA and 0.75 mL EDA were dissolved into 50 mL water and sonicated for 10 min. Then the solution was transferred into a Teflon-lined autoclave and heated at 200 ℃ for 8 h. After natural cooling down, the crude product was filtered through 0.2 μm syringe filter. The filtrate was stored as stock solution for further purification. The as-obtained CDs were named CA-EDA CDs and abbreviated as CE-CDs in the subsequent sections. For CA-PPD-derived CDs (CP-CDs), 0.75 mL EDA was replaced with 1.22 g PPD, while all treatments were the same of CE-CDs.

For further purification of CDs, dialysis was selected. As pre-filtration removed larger impurities, dialysis could help to eliminate smaller molecular and oligomeric impurities. For both CE-CDs and CP-CDs, the stock solutions were aliquoted into 5 mL tubes and submitted to dialysis. Dialysis membranes with molecular weight cut-off 1000 Da were used. During the dialysis, the water was changed seven times a day, at 8 am, 10 am, 12 pm, 2 pm, 4 pm, 6 pm, and 8 pm. The volume ratio of filtrate to water was 1:100 to ensure a fast exchange process. Retentates after dialysis of 1, 2, 4, 8, 12, 24, 36, 48, 72, 96, 120, 144, 168, and 240 h were collected and lyophilized for further characterizations.

*Characterizations*

The morphologies of CE-CDs and CP-CDs were characterized using transmission electron microscopy (Hitachi 7500). UV-Vis absorption and fluorescence emission spectra were recorded by using a UV-Vis spectrometer (Cary 5000) and fluorometer (JACSO FP-8300) in deionized water at a concentration of 10 μg/mL. Fourier-transform infrared (FTIR) spectra were recorded by using an FTIR spectrometer (JACSO FTIR-4100). NMR spectra were collected using NMR spectrometers (Bruker Avance 500 MHz and Ascend 600 MHz) at a sample concentration of 20 mg/mL. X-ray photoelectron spectroscopy (XPS) was conducted by a multiprobe system (Thermo Scientific) equipped with an Al Kα source (1486 eV).


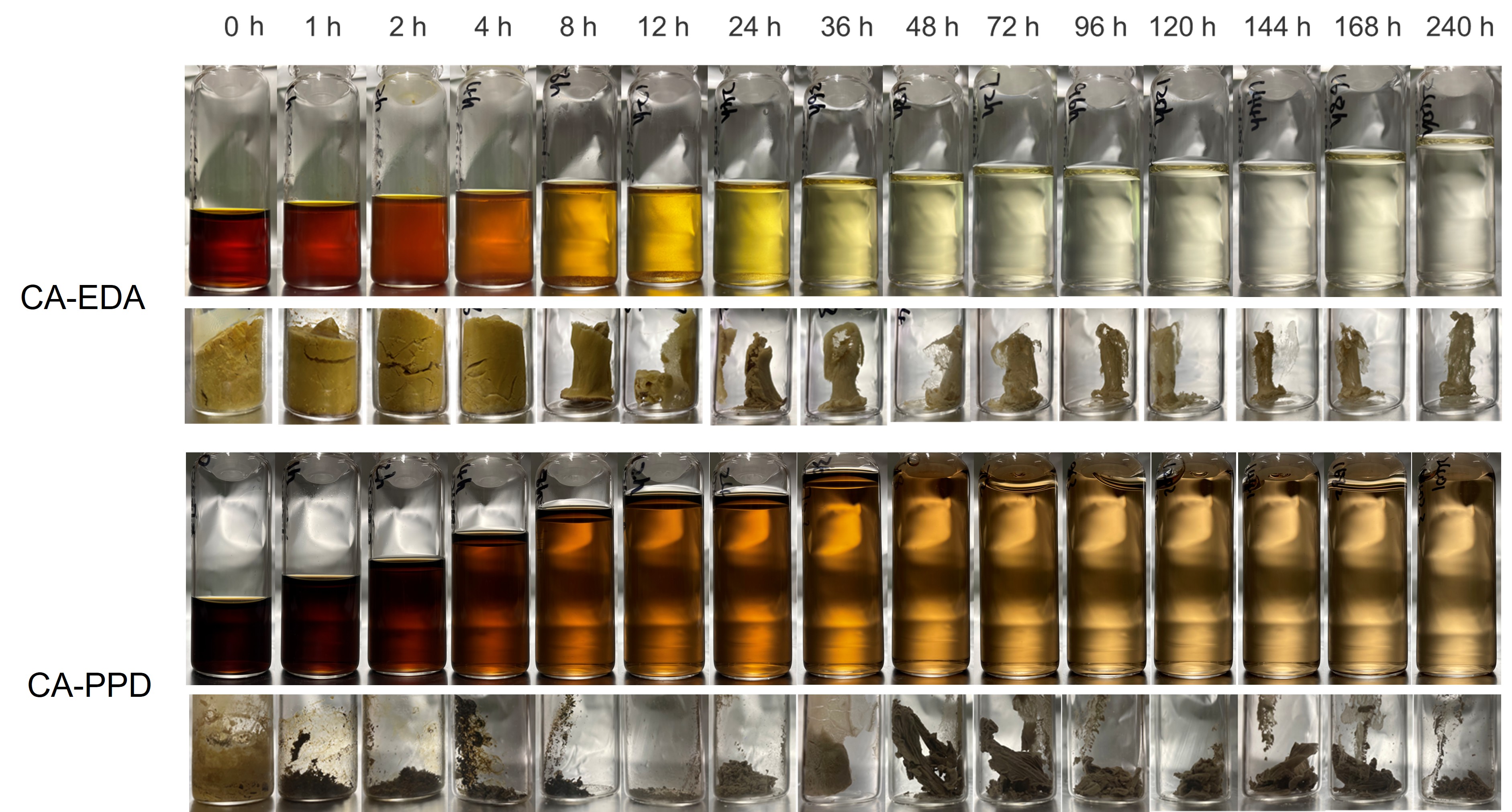


**Figure S1**. Photographs of liquid and solid CE-CDs and CP-CDs retentates under visible light.


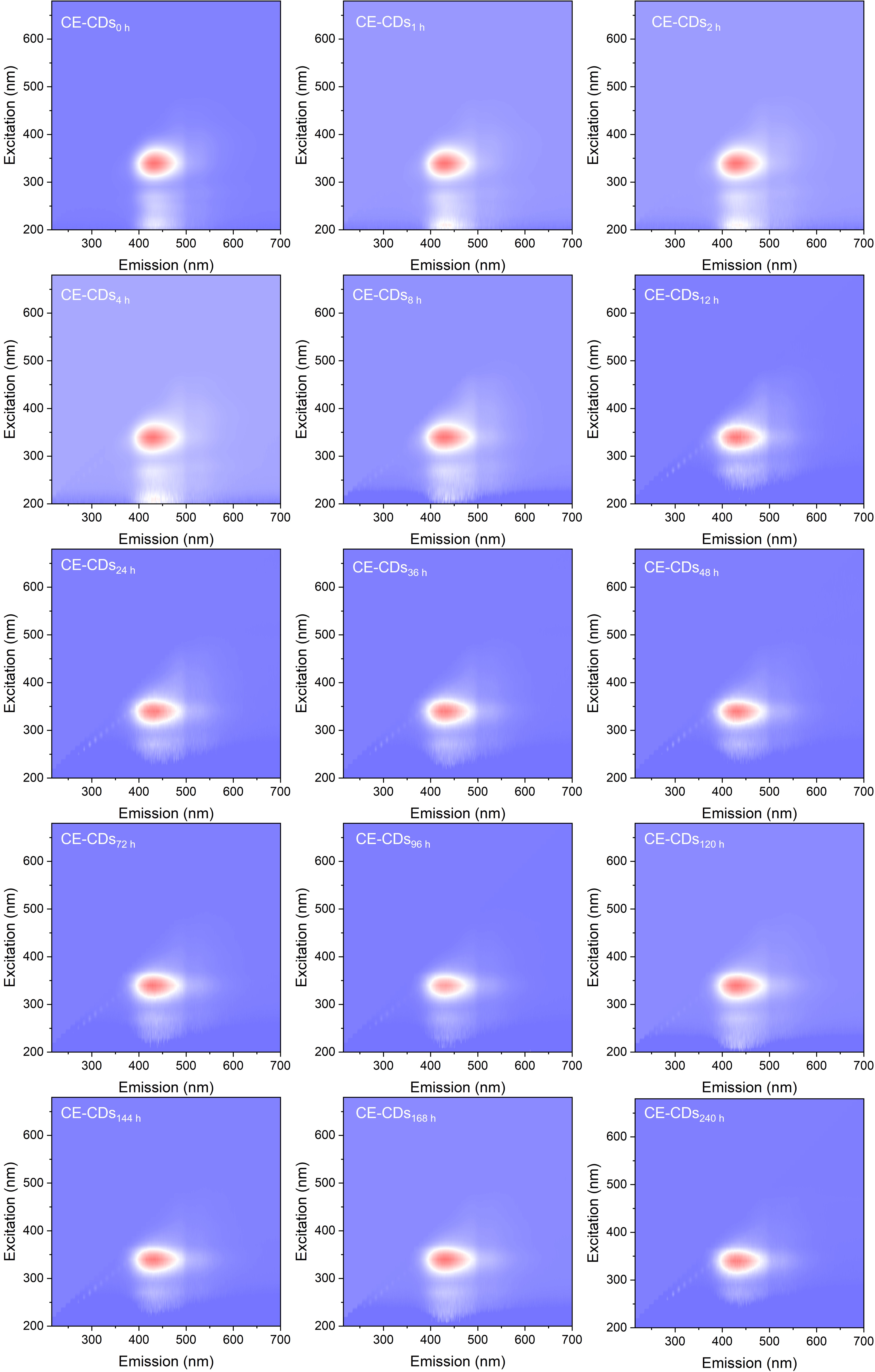


**Figure S2**. Fluorescence excitation-emission matrices of CE-CDs retentates.


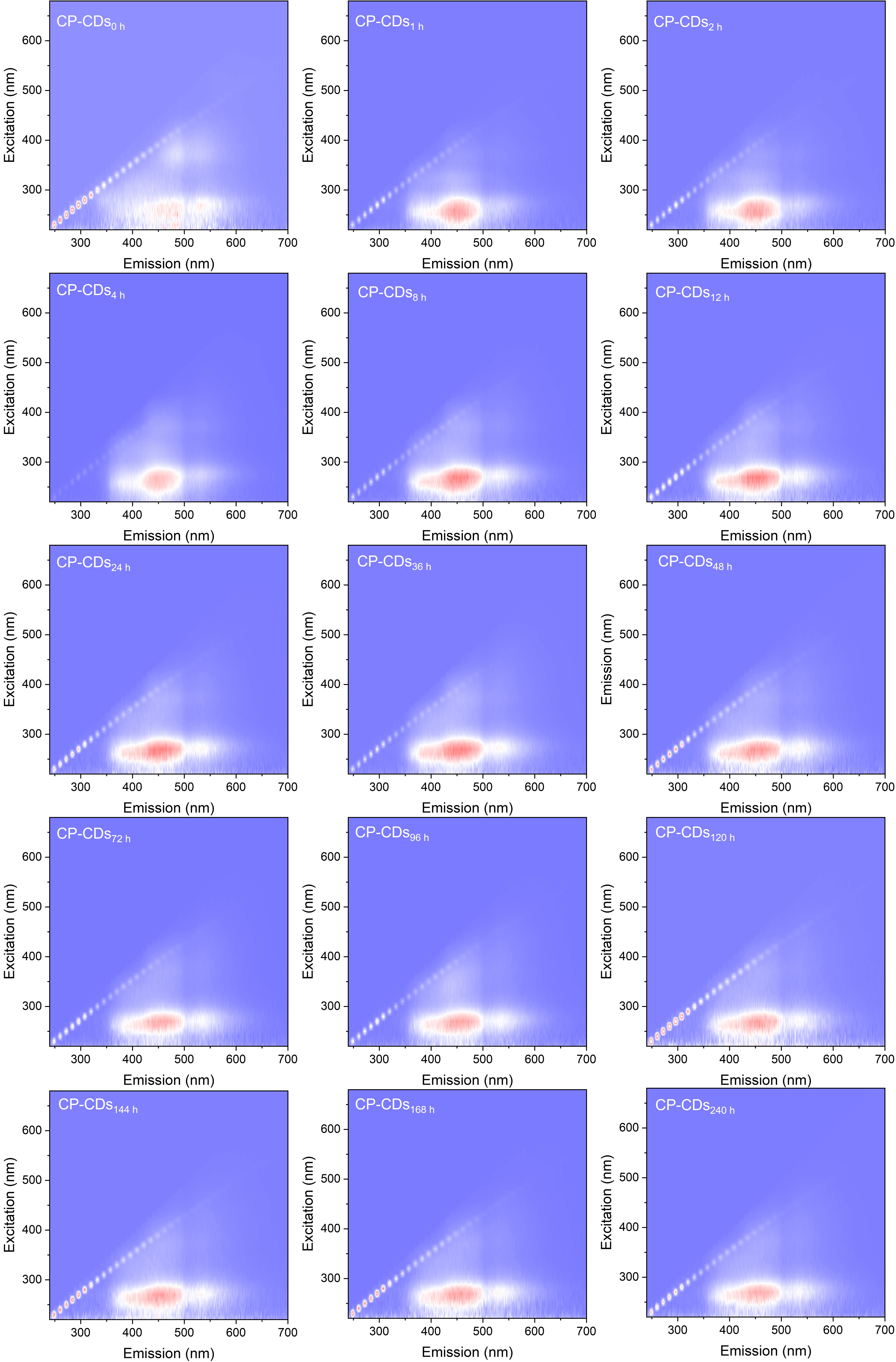


**Figure S3**. Fluorescence excitation-emission matrices of CP-CDs retentates.


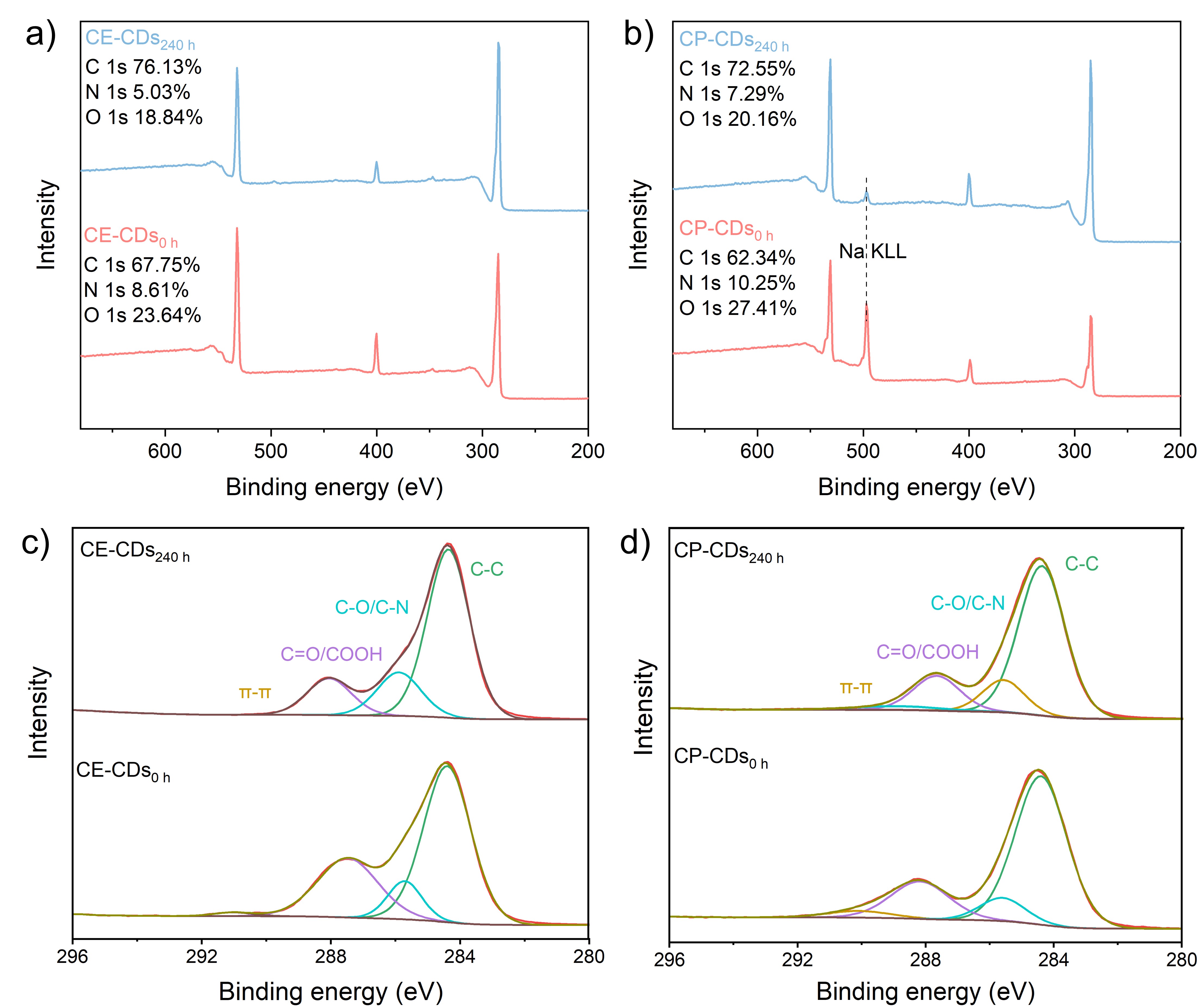


**Figure S4**. XPS survey spectra of CE-CDs (a) and CP-CDs (b) before and after 240 h dialysis; High-resolution C 1s spectra of CE-CDs (c) and CP-CDs (d) before and after 240 h dialysis.


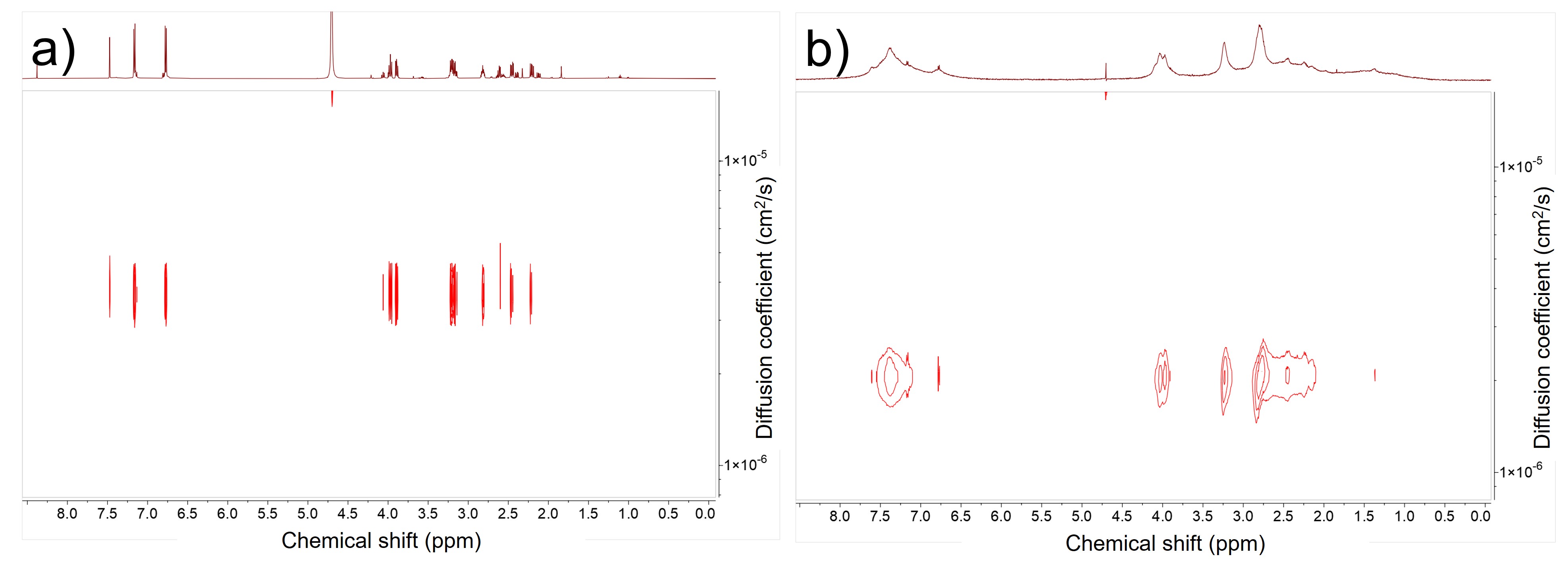


**Figure S5**. DOSY spectra in D_2_O of CP-CDs retentates at 0 h (a) and 240 h (b).


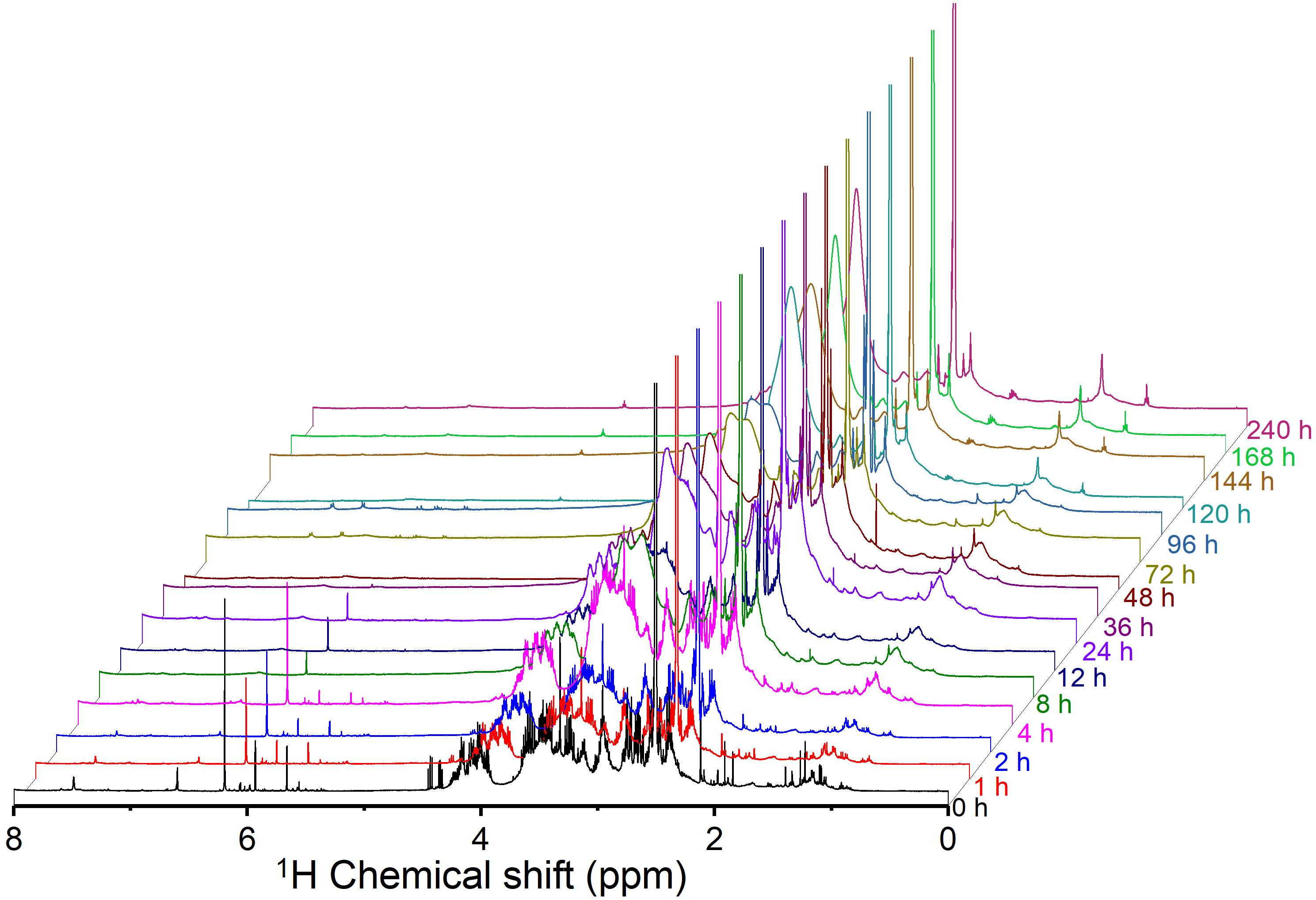


**Figure S6**. NMR spectra of CE-CDs retentates in DMSO-*d6*.


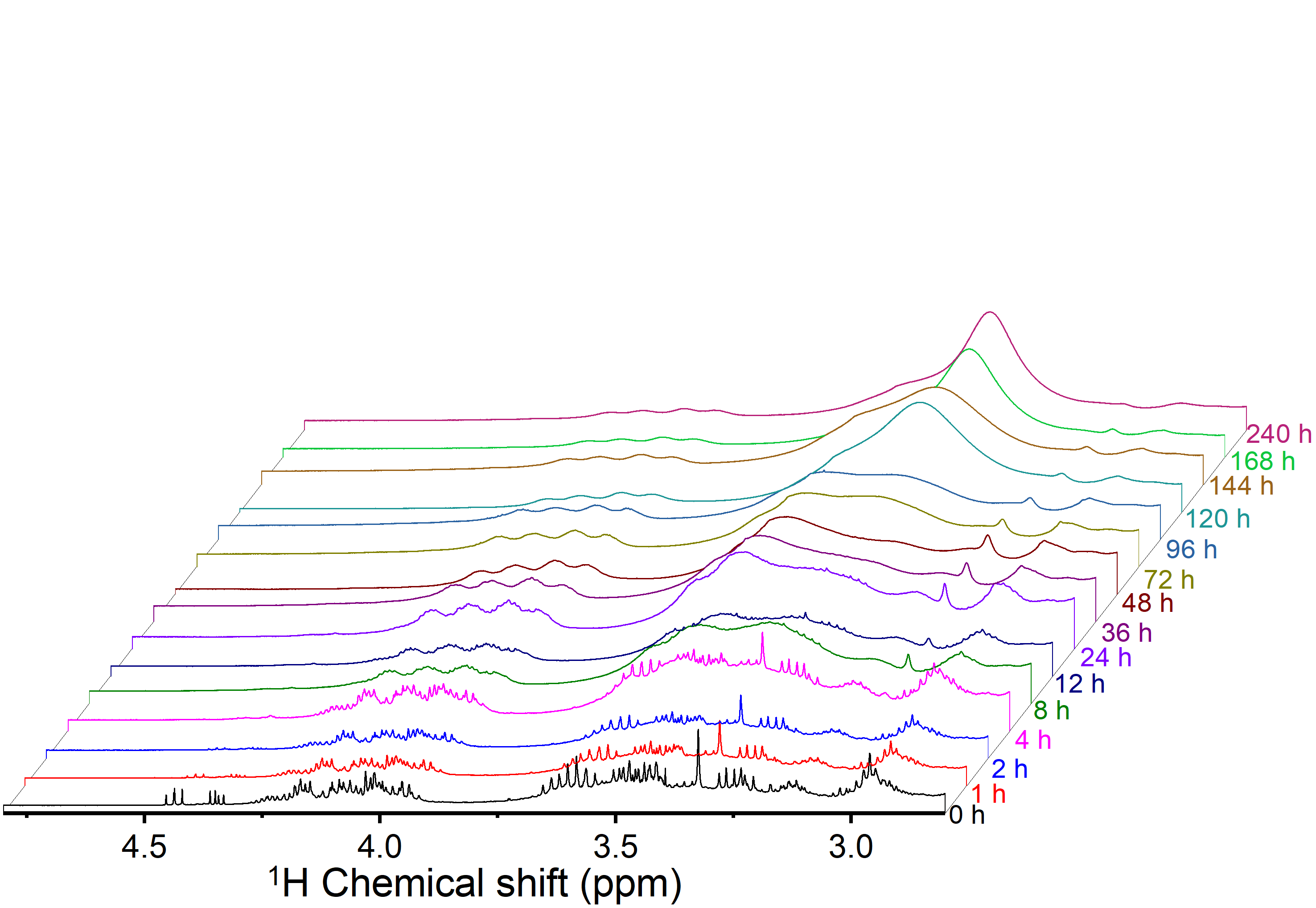


**Figure S7**. Zoom NMR spectra of CE-CDs retentates in DMSO-*d6*.


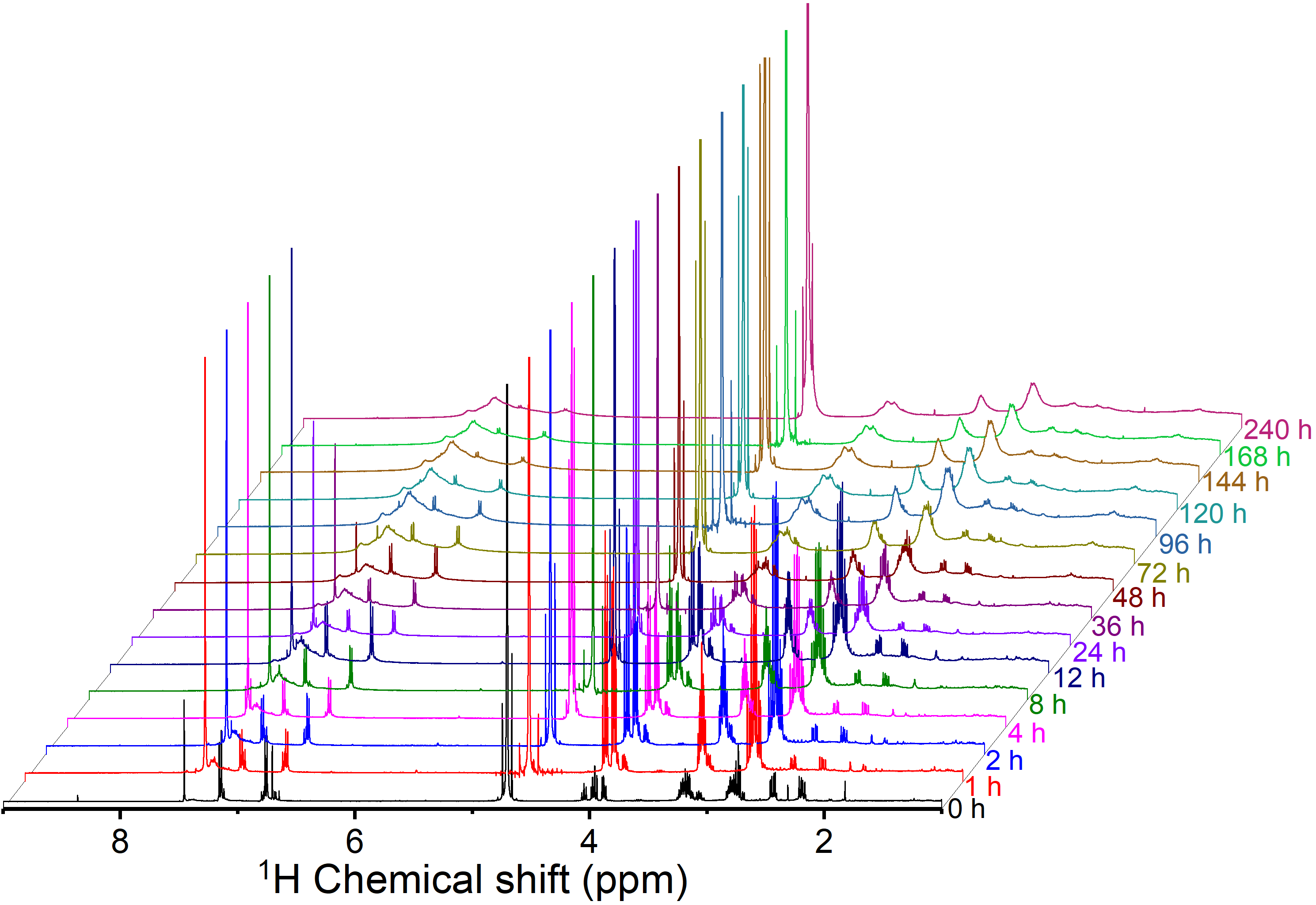


**Figure S8**. NMR spectra of CP-CDs retentates in D_2_O.


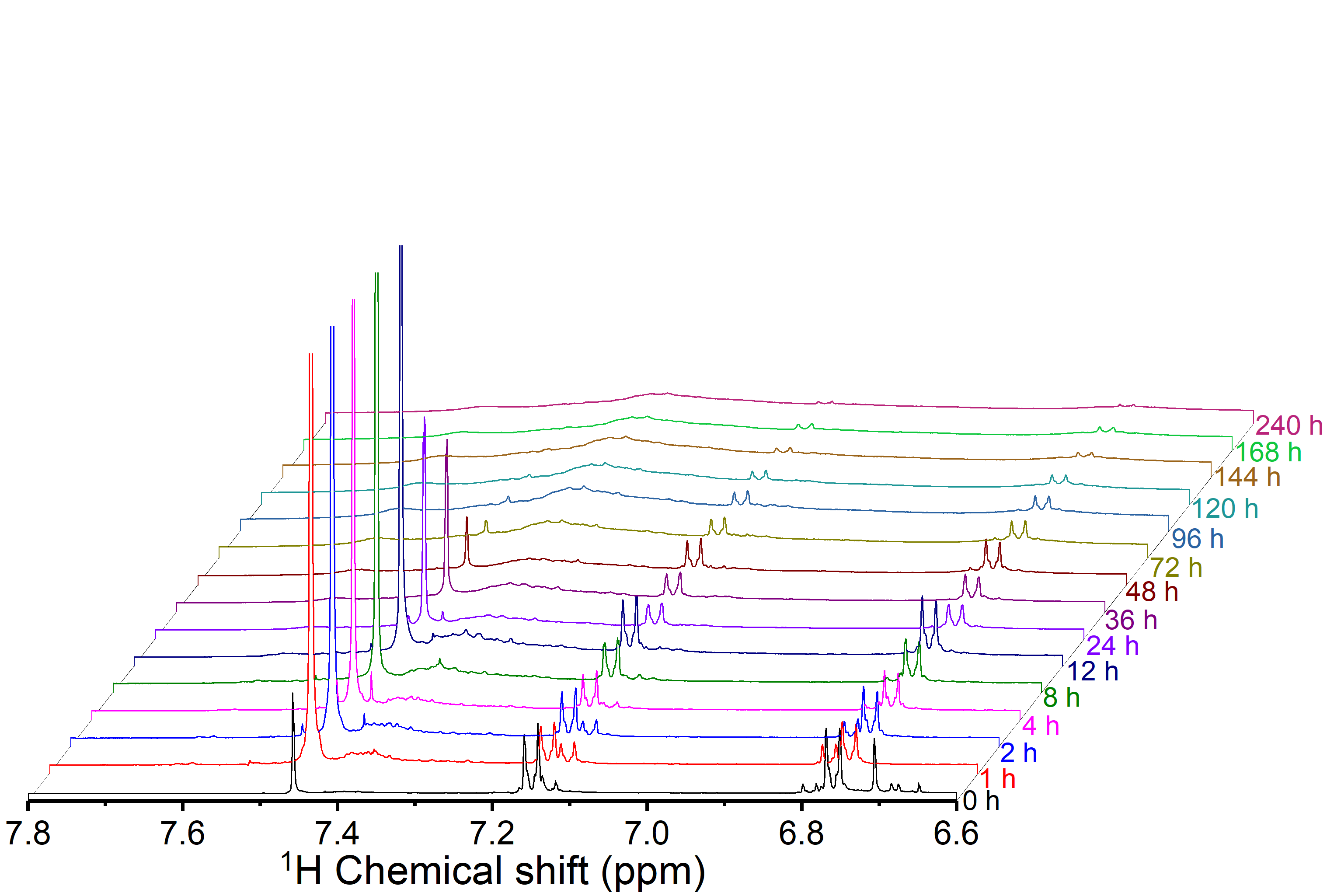


**Figure S9**. Zoom NMR spectra of CP-CDs retentates in D_2_O.


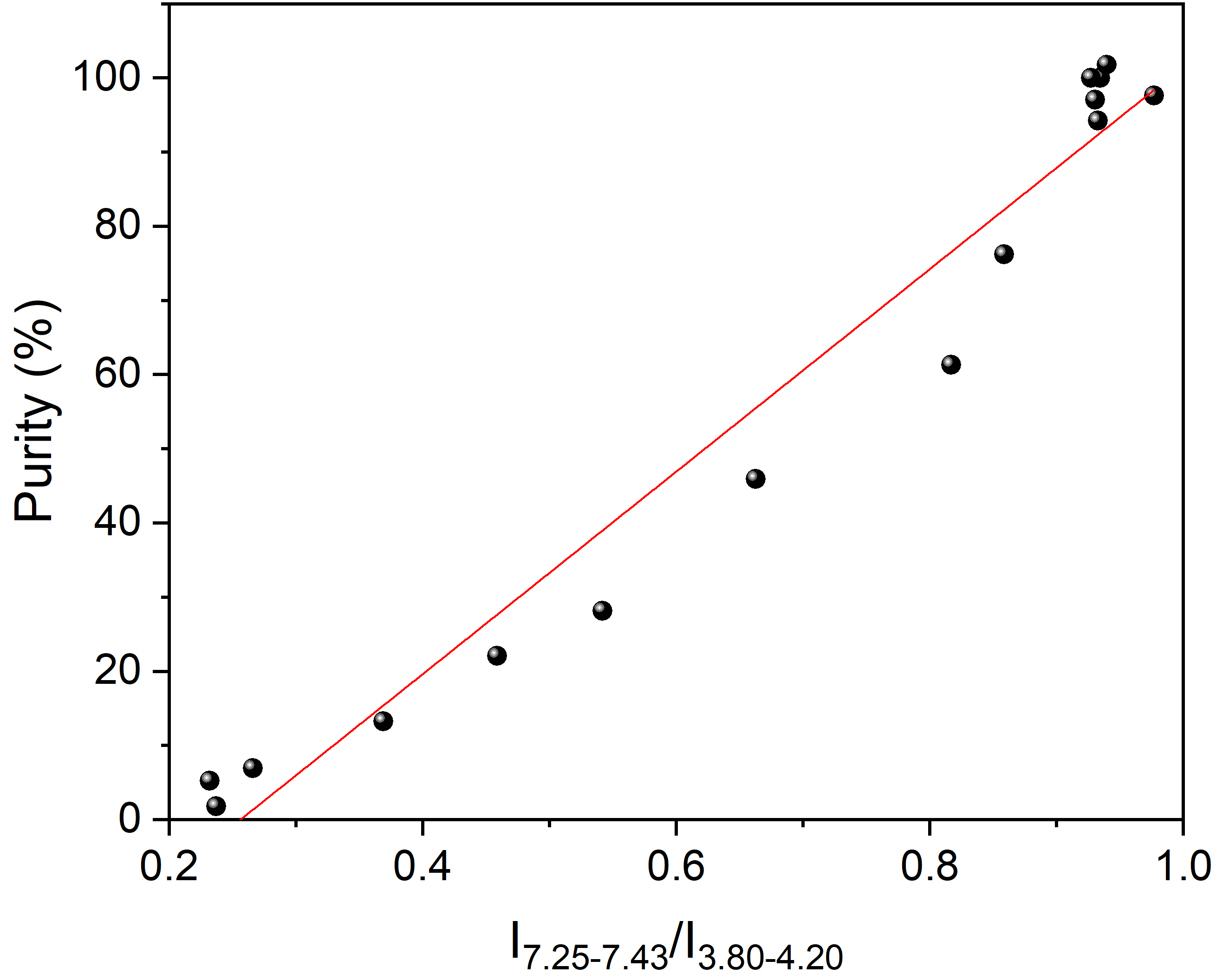


**Figure S10**. Purity of CP-CDs retentates versus I_7.25-7.43_/I_3.80-4.20_.
